# 11C-choline PET/CT and whole-body MRI including diffusion-weighted imaging for patients with recurrent prostate cancer

**DOI:** 10.18632/oncotarget.16227

**Published:** 2017-03-15

**Authors:** Hinrich Wieder, Ambros J. Beer, Konstantin Holzapfel, Martin Henninger, Tobias Maurer, Sarah Schwarzenboeck, Ernst J. Rummeny, Matthias Eiber, Jens Stollfuss

**Affiliations:** ^1^ Department of Nuclear Medicine, Klinikum rechts der Isar, Technische Universität München, Munich, Germany; ^2^ Centre for Radiology and Nuclear Medicine, Grevenbroich, Germany; ^3^ Institute for Diagnostic and Interventional Radiology, Klinikum rechts der Isar, Technische Universität München, Munich, Germany; ^4^ Department of Urology, Klinikum rechts der Isar, Technische Universität München, Munich, Germany; ^5^ Department of Nuclear Medicine, Universitätsmedizin Rostock, Rostock, Germany; ^6^ Department of Radiology and Nuclear Medicine, Klinikum Memmingen, Memmingen, Germany; ^7^ Department of Nuclear Medicine, Ulm University, Ulm, Germany

**Keywords:** prostate cancer, recurrence, 11C-choline, PET/CT, MRI

## Abstract

**Purpose:**

To compare the detection efficacy of 11C-choline positron emission tomography and computed tomography (PET/CT) with whole-body magnetic resonance imaging (MRI) including diffusion-weighted imaging (DWI) in patients with suspected recurrent prostate cancer.

**Materials and Methods:**

Fifty-seven patients (mean age 68, range 54-80 years) underwent 11C-choline PET/CT and MRI using T1-weighted (T1w), short-tau inversion recovery (STIR), and DWI. Two readers visually rated suspicious lesions on a 5-point scale in 20 different regions. Clinical follow-up and histopathology served as the standard of reference (SOR).

**Results:**

Fifty patients (mean PSA 29.9, range 1.0-670 ng/mL) had at least one positive lesion according to the SOR. Twenty-four patients had local recurrence (LR), 27 had lymph node (LN) involvement, and 22 had bone metastases. The overall detection rates for PET/CT and MRI on a patient basis were 94% and 88%, respectively (*p* = 0.07). The PSA level (>2 ng/mL vs ≤2 ng/mL) significantly influenced the overall performance of PET/CT (*p* = 0.003) and MRI (*p* = 0.03). PET/CT was significantly superior to MRI in detecting LR (*p* = 0.03) and bone metastasis (*p* = 0.02). We found no difference with respect to the detection of LN metastasis (*p* = 0.65).

**Conclusion:**

11C-choline PET/CT was superior in the detection of local recurrence and bone metastasis on a regional basis. Whole-body MRI including DWI showed similar diagnostic accuracy only for detecting lymph node metastases. Compared with 11C-choline PET/CT, therefore, whole-body MRI including DWI cannot serve as alternative imaging modality for restaging prostate cancer.

## INTRODUCTION

Prostate cancer is the third most common cause of death from cancer in men (9.5%) in Europe and is associated with a high rate of recurrence [1]. After primary treatment, relapse occurs in 20-30% of patients within 10 years of prostatectomy [2] and in up to 53% within 5 years of external-beam radiotherapy [3]. The diagnostic sensitivity of conventional imaging in the setting of PSA recurrence is often disappointing.

Positron emission tomography (PET) using 11C-choline has been shown to be promising in both staging and follow-up of patients with prostate cancer [4, 5]. PET is usually performed in combination with computed tomography (PET/CT), which provides a comprehensive anatomical and molecular whole-body survey in a single imaging session [6]. In the case of recurrent prostate cancer, combining the two modalities facilitates the anatomical localization of PET-positive findings [7]. However, C11-cholin fails to identify a positive lesion in a substantial number of cases, especially in patients with PSA values lower than 2 ng/mL. However, initial PET studies using Prostate-specific membrane antigen ligands (e.g. 68Ga-PSMA HBED-CC) recently achieved superior detection rates compared to 11C-choline even at low PSA [8].

Whole-body magnetic resonance imaging (MRI) became possible with the development of parallel imaging and high-gradient amplitudes [9, 10]. High spatial resolution diffusion-weighted imaging (DWI) has also been introduced [11]. This technique offers high lesion-to-background contrast and has been suggested as an adjunct to detect primary malignancies and metastases in oncology patients [12-14]. DWI may also be used to characterize lesions by quantitative apparent diffusion coefficient (ADC) values. ADC values have been regarded as a potential imaging biomarker comparable to standardized uptake values (SUV) in PET/CT [15]. Preliminary data have shown an incremental value of DWI for characterizing pelvic lymph nodes and bone metastases in prostate cancer [7, 16]. However, despite the increasing use of DWI in patients with cancer, systematic reports evaluating this technique within the concept of whole-body MRI are still limited [11, 13, 14, 17, 18].

The aim of our study, therefore, was to compare the detection rate of 11C-choline PET/CT with that of whole-body MRI including DWI in patients with suspected recurrent prostate cancer.

## RESULTS

### SOR

In total, 1026 regions (57 LR, 456 LN, 456 bone, 57 other) were evaluated in 57 patients. Fifty patients had at least one positive lesion according to the SOR, 153 regions were finally regarded as positive, and 873 as negative. Twenty-four of the 57 patients included in the study showed evidence of local recurrence. Of 456 lymph node regions evaluated in the study, 59 regions in 27 patients were regarded as positive, 397 regions in 30 patients as negative. Seventy of the 456 bone regions in 22 patients were regarded as positive and 386 regions in 35 patients as negative. Seventeen of 57 patients had evidence of extra-pelvic disease. Five patients had metastases in other regions (lung in four cases, liver in two cases, and peritoneum in one case). These lesions were excluded from analysis.

### Detection rate on a patient basis

11C-choline PET/CT showed positive results in 94.0% (47/50 patients). MRI analysis resulted in a detection rate of 88.0% (44/50 patients). The PAC of both methods was significantly higher in the subgroup of patients with PSA values greater than 2 ng/mL compared with the subgroup with values lower than or equal to 2 ng/mL (Table 2). However, there was no significant difference between the methods on a patient basis. Both, PET/CT and MRI correctly identified all patients with evidence of extra-pelvic disease (showing at least one positive extra-pelvic lesion per patient). For 11C-Choline PET/CT mean imaging time was calculated as 18.4 min ± 0.7 min (range 17 - 21 min). For 11C-Choline PET/MR mean imaging time was 50.4 min ± 7.9 min (range 42 - 92 min).

**Table 1:** Patient characteristics

Number of patients	57
Primary radical prostatectomy	100%
Age [years]	
Median (range)	86 (54-80)
Gleason score *	
mean (range)	8 (6-9)
PSA [ng/ml]	
mean (range)	29.9 (1.0 – 670)
Patients with PSA > 2.0 [ng/ml] mean PSA±SD [ng/ml]	42 (74%) 16.0 ± 33.4
Patients with PSA ≤ 2.0 [ng/ml] mean PSA±SD [ng/ml]	15 (26%) 1.4 ± 0.3
Interval: primary treatment – 11C-choline PET/CT-imaging [months]	
median (range)	77 (6-200)

* Information about the initial Gleason score could be obtained in 42 of the 57 patients.

**Table 2:** Overall accuracy of PET/CT and MRI for detection of local recurrence and/or metastases on a patient basis.

PAC	PET/CT	MRI
All	95.0 % *	88.0 % *
PSA >2 ng/ml	100.0 % # †	90.5 % ¶ †
PSA ≤ 2 ng/ml	80.0 % # ††	66.7 % ¶ ††

The comparison of PET/CT and MRI showed no significant difference in overall predictive accuracy (PAC) on a patient basis (*p = 0.07). However, the PAC was significantly higher in the subgroup of patients with PSA level >2 ng/mL than in patients with PSA level ≤ 2 ng/mL for both PET/CT and MRI (# *p* = 0.003; ¶ *p* = 0.03). Comparing PET/CT and MRI in the subgroups, PET/CT was significantly superior to MRI in patients with PSA level >2 ng/mL († p=0.04) but not in the subgroup of patients with PSA level ≤ 2 ng/mL (†† *p* = 0.41).

### Regional lesion detection

The sensitivity and overall PAC of 11C-choline PET/CT and PET/MR in detecting LR, LN and bone metastases on the basis of the dichotomized data is given in Table 3. In keeping with the results of the patient based evaluation, analysis of all 3 regions generally showed higher sensitivities in all patients and in the subgroup of patients with PSA values >2 ng/mL compared to patients with PSA values ≤2 ng/mL.

**Table 3:** Accuracy of PET/CT and MRI in detecting local recurrence, lymph node metastases, and bone metastases

	LR		LN		Bone	
	PET/CT	MRI	PET/CT	MRI	PET/CT	MRI
**All patients**						
Sensitivity	83.3% (20/24)*	54.2% (13/24)*	81.4% (48/59) ¶	77.9% (46/59) ¶	92.9% (65/70) ¶¶	78.6% (55/70) ¶¶
Specificity	93.9% (31/33)	81.8% (27/33)	99.7% (396/397)	87.5% (356/407)	98.4% (380/386)	87.5% (356/407)
PPV	90.9% (20/22)	68.4% (13/19)	97.9% (48/49)	52.8% (46/87)	91.5% (65/71)	52.9% (46/87)
NPV	88.6% (31/35)	71.1% (27/38)	97.3% (396/407)	96.5% (356/369)	98.7% (380/385)	96.5% (356/369)
PAC	89.5% (51/57)	70.1% (40/57)	97.4% (444/456)	88.2% (402/456)	97.6% (385/456)	88.2% (402/456)
**PSA > 2 ng/ml**						
Sensitivity	90.5% (19/21) †	52.4% (11/21) †	90.5% (38/42) §	83.3% (35/42) §	95.1% (58/61) #	80.3% (49/61) #
Specificity	90.5% (19/21)	76.2% (16/21)	99.7% (293/294)	89.0% (260/292)	98.4% (380/386)	97.1% (267/275)
PPV	90.5% (19/21)	68.8% (11/16)	97.4% (38/39)	52.2% (35/67)	90.3% (65/72)	85.9% (49/57)
NPV	90.5% (19/21)	61.5% (16/26)	98.7% (293/297)	97.4% (260/267)	98.7% (380/385)	95.7% (267/279)
PAC	90.5% (38/42)	88.1% (37/42)	98.5% (331/336)	87.8% (295/336)	97.6% (445/456)	94.0% (316/336)
**PSA ≤ 2 ng/ml**						
Sensitivity	33.3% (1/3) ††	66.6% (2/3) ††	58.8% (10/17) §§	64.7% (11/17) §§	77.8% (7/9) ##	66.7% (6/9) ##
Specificity	100% (12/12)	91.7% (11/12)	100% (103/103)	91.4% (96/105)	98.2% (109/111)	95.5% (106/111)
PPV	100% (1/1)	66.7% (2/3)	100% (10/10)	55.0% (11/20)	77.8% (7/9)	55.5% (6/11)
NPV	85.7% (12/14)	91.7% (11/12)	93.6% (103/110)	94.1% (96/102)	98.2% (109/111)	97.2% (106/109)
PAC	86.7% (13/15)	86.7% (13/15)	94.2% (113/120)	89.2% (107/120)	96.7% (111/120)	93.3% (112/120)

Considering all patients, PET/CT showed a significantly higher PAC than MRI for the detection of both local recurrence (LR) (*p = 0.03) and bone metastases (Bone) (¶¶ *p* = 0.02). Comparison of PET/CT and MRI on the basis of regions showed no significant difference in the sensitivity of the detection of lymph node (LN) metastasis (¶ *p* = 0.65). Subgroup analysis of patients with PSA >2 ng/mL yielded similar results († *p* = 0.006; § *p* = 0.33; # *p* = 0.01). However, the regional differences were generally not significant for patients with PSA ≤ 2 ng/mL, probably owing to the relatively low number of local recurrences and bone metastases in this subgroup (†† *p* = 0.85; §§ *p* = 0.72; ## *p* = 0.59).

Regional analysis of all patients also revealed a significantly higher sensitivity of 11C-choline PET/CT compared to MRI for the detection of LR and bone metastases. However, no significant difference was found with respect to LN metastasis. Cohen’s kappa analysis showed only slight agreement between PET/CT and MRI for the detection of LR (kappa 0.188, 0.027-0.349), fair agreement for LN metastases (kappa 0.349, 0.256-0.442), and moderate agreement for bone metastasis (kappa 0.582, 0.490-0.674). Both 11C-choline PET/CT and MRI generally showed a random distribution of false positives and negatives over the LN and bone regions evaluated. However, none of the false negative bone regions on MRI (*n* = 15) was located in the cervical, thoracic or lumbar spine (3 in the sacrum, 8 in the pelvis, and 4 in the extremities).

In the subgroup of patients with PSA values >2 ng/mL, 11C-choline PET/CT again showed significantly greater efficiency than MRI in detecting LR and bone metastasis, and equal results for the detection of LN metastasis. Differences between the two methods were also apparent in the subgroup of patients with PSA values ≤2 ng/mL but failed to reach the level of statistical significance.

**Figure 1:**
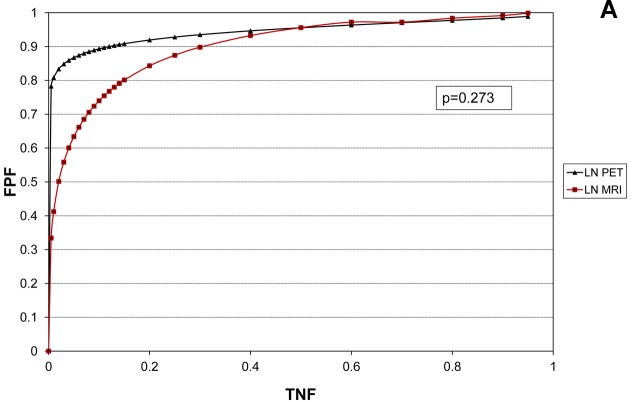

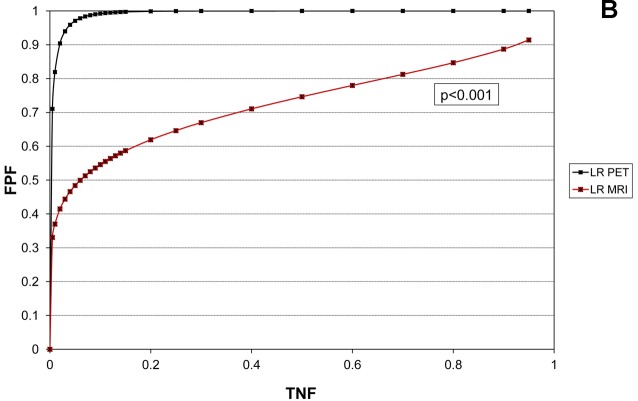

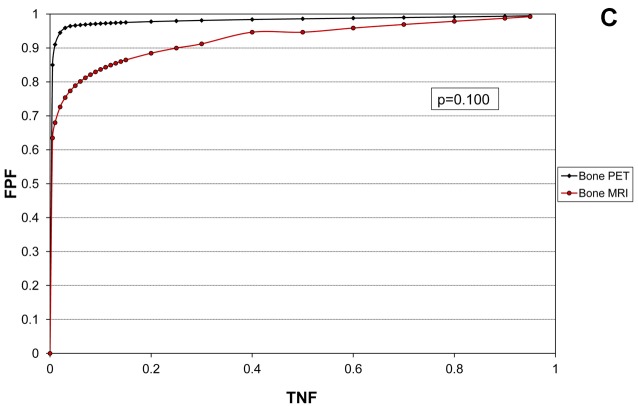
Considering all patients, ROC analysis with areas under the curve (AUC) shows no significant differences for the detection of lymph node (LN, **A**) metastasis. PET/CT showed significantly greater AUCs than MRI for the detection of both local recurrence (LR, B) and bone metastases (Bone, C) (see also Table 4).

Table 4 shows the data on the diagnostic performance using ROC analysis. The ROC graphs separated for LR, LN, and bone metastases are shown in Figure 1a-1c.

**Table 4:** Comparison of diagnostic performance by ROC analysis

**AUC**	**PET/CT**	**MRI**	***p*-value**
**All patients**			
LR (*n* = 57)	0.993 (0.946-0.999)	0.729 (0.555-0.860)	*P* = 0.001
LN (*n* = 456)	0.945 (0.850-0.985)	0.905 (0.849-0.944)	*P* = 0.273
Bone (*n* = 456)	0.984 (0.900-0.998)	0.925 (0.893-0.971)	*P* = 0.100
**PSA >2 ng/ml**			
LR (*n* = 42)	0.979 (0.888-0.998)	0.720 (0.539-0.856)	*P* = 0.002
LN (*n* = 336)	0.941 (0.802-0.989)	0.922 (0.861-0.960)	*P* = 0.653
Bone (*n* = 336)	0.982 (0.888-0.999)	0.923 (0.816-0.974)	*P* = 0.164
**PSA ≤2 ng/ml**			
LR (*n* = 15)	0.908 (0.613-0.991)	0.632 (0.189-0.941)	*P* = 0.149
LN (*n* = 120)	0.903 (0.697-0.981)	0.775 (0.512-0.931)	*P* = 0.264
Bone (*n* = 120)	0.977 (0.899-0.997)	0.928 (0.736-0.989)	*P* = 0.309

Areas under the curve (AUC) with 95% confidence intervals given in brackets. Considering all patients, the ROC analyses showed no significant differences in AUCs for the detection of lymph node metastasis (LN). Compared with MRI, PET/CT showed significantly greater AUCs in the detection of both local recurrence (LR) and bone metastasis (Bone). Subgroup analysis of patients with PSA >2 ng/mL showed a significant difference for the detection of local recurrence. Regional differences were generally not significant for patients with PSA ≤ 2 ng/mL.

## DISCUSSION

Approximately one-third of patients with prostate cancer will have a recurrence of disease after definitive treatment with surgery or external radiation [19]. In most patients, recurrence is suspected because of an increase in PSA levels, which may precede clinically detectable disease by months or even years. Traditionally, screening for distant metastasis requires a combination of radionuclide bone scanning, CT, and MRI. Each of these techniques has its limitations and an accurate non-invasive method for guiding pathological confirmation on a whole-body basis is greatly to be desired [20].

We set out to determine the diagnostic performance of 11C-choline PET/CT in detecting recurrent disease in patients with prostate cancer, and to compare it with that of whole-body MRI including DWI. The overall detection rate for 11C-Choline PET/CT was higher compared to a study of Castellucci and colleagues (about 39%) probably owing to the high PSA level in our study cohort [15]. However, the detection rate of MRI with DWI was lower than we might have expected from studies using DWI or contrast enhanced MRI protocols [21, 22]. Both, 11C-choline PET/CT and MRI showed variable diagnostic accuracy for detecting LR, LN metastases, and bone metastases on a regional basis. The efficacy of 11C-choline PET/CT in detecting LR and bone metastases was clearly better than that of MRI including DWI. With respect to the detection of LN metastases, we did not find a significant difference in the accuracy of the two methods. The PSA level significantly influenced the overall accuracy of both PET/CT and MRI. This is in agreement with the findings of other studies that have investigated the dependence of 11C-choline PET/CT detection on PSA values in the setting of biochemical recurrence [15, 23].

Lesions to the lungs and visceral organs were relatively infrequent in our study. However, we excluded those from analysis because of inherent limitations of both MRI (concerning the lung lesions) and PET (concerning liver lesions). Compared to PET/CT, the MRI acquisition time was significantly longer which represents a potential limitation regarding patient throughput. Ten patients had to be excluded from the study because of general MRI contraindications also representing a limitation of this imaging method.

### Local recurrence

However, the diagnosis of LR after radical prostatectomy is often challenging. Souvatzoglou et al. found that only seven (19%) of 37 patients with low PSA values (PSA range, 0.3-1.8 ng/mL) who were referred for salvage radiotherapy showed an increased uptake in the prostatectomy bed using 11C-choline PET/CT [24]. Panebianco et al. compared multiparametric MRI and 18F-choline PET/CT for detecting LR after radical prostatectomy in 84 patients and showed MRI to be superior, notably in patients with smaller lesions (< 7mm) and low PSA values [21]. However, their MRI protocol included dedicated sequences for the pelvis, e.g. dynamic contrast enhanced (DCE) imaging, which we did not use in our study so as to stay within a reasonable time frame for whole-body imaging. In addition, the limited number of patients with PSA values < 2 ng/mL in our study might have contributed to results in favour of 11C-choline PET/CT. A recent study on whole-body MRI in combination with dedicated MR for the Prostate showed disease recurrence in only 21% (16/76) of PC patients having with median PSA of 0.36 ng/mL (range 0.05-56.12) [25].

**Figure 2:**
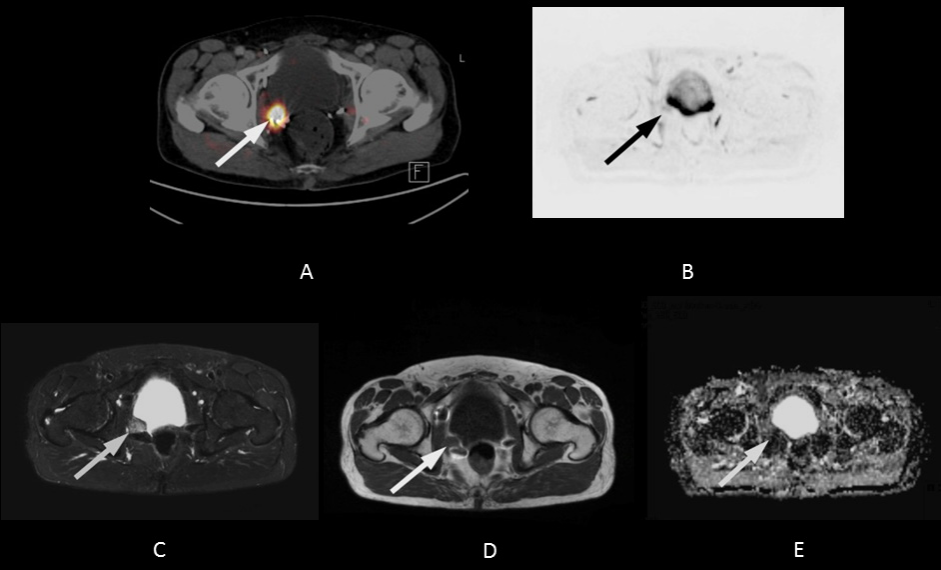
68-year-old patient with local recurrence 13 years after radical prostatectomy (initial Gleason Score: 8) The PSA level at the time of imaging was 2.1 ng/mL. A fall in PSA level after pelvic irradiation confirmed the lesion was malignant (< 1.0 ng/mL after radiotherapy). **A.** The axial PET/CT fusion image shows focal 11C-choline uptake in the area of the right neurovascular bundle (white arrow, SUV max 4.2). **B.** The lesion was not visible on the 500 ms b-image (B) or the ADC map (not shown) of the DWI. Both the STIR image **C.** and the T1w image **D.** show susceptibility artefacts due to surgical clips in the area of focal 11C-choline uptake. Part of the lesion, however, remains visible in spite of some signal loss (white arrows).

A wide variety of entities can mimic recurrent prostate cancer on MRI. Retained seminal vesicles, fibrotic scaring, a prominent peri-prostatic venous plexus, and granulation tissue can all lead to false positive results [26]. In addition, susceptibility artefacts due to surgical paramagnetic material may also diminish the accuracy of MRI in the detection of LR (Figure 2).

### Lymph nodes

**Figure 3:**
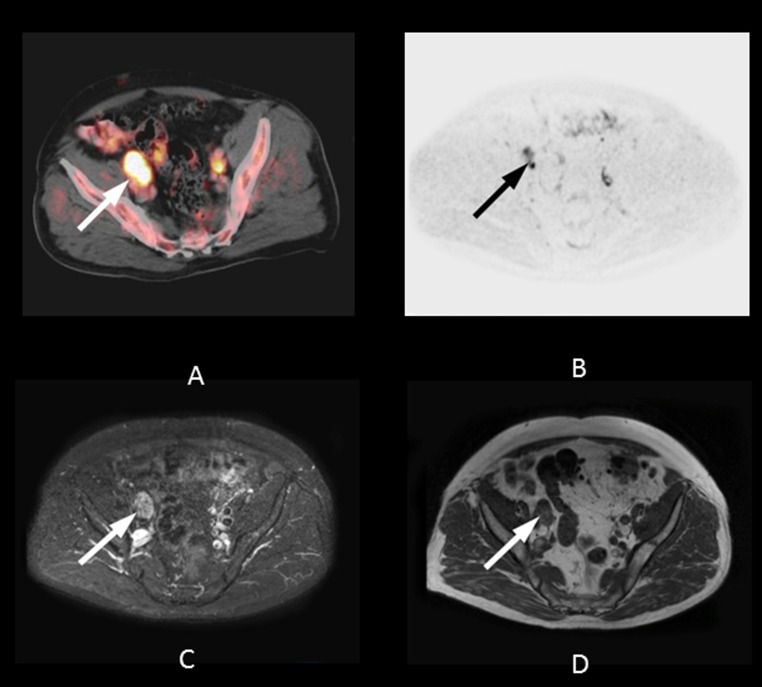
74-year-old patient with lymph node metastases six years after radical prostatectomy (initial Gleason Score: 7) The PSA level at the time of imaging was 21.0 ng/mL. A regression in lesion size after pelvic irradiation confirmed the malignant nature of the lesions. **A.** Axial PET/CT fusion image showing focal 11C-choline uptake in the area of the right external iliac vessels (white arrow, SUV max 3.6). The lesion shows focal signal elevation on both the 500 ms b-image using DWI **B.** (black arrow) and the STIR image **C.** (white arrow). **D.** Axial fusion T1w image showing enlarged lymph nodes (20 mm) close to the right external iliac vein.

The evaluation of LN metastases is crucial in restaging patients with PSA failure after radical prostatectomy. Recent studies have evaluated the diagnostic performance of MRI with DWI and 11C-choline PET/CT in preoperative lymph node staging, yielding varying results. In a patient-based analysis of 132 patients, Beheshti et al. obtained a sensitivity of 66% and specificity of 96% in lymph nodes larger than 5 mm when using 11C-choline PET/CT [27]. In a cohort of 29 patients, Eiber et al. calculated a sensitivity of 86% and specificity of 85.3% for DWI of pelvic lymph nodes in prostate cancer [28]. In contrast, Budiharto et al. reported disappointing results in a group of 36 high-risk prostate cancer patients [29]. The sensitivity in a per-patient analysis was only 43% with a specificity of 98%. We found equal diagnostic accuracy for 11C-choline PET/CT and MRI. The relatively high overall detection rate for both methods in our study might be due to summarizing multiple nodes to regions, in contrast to a true lesion-by-lesion analysis based on histopathology, as performed in other investigations (Figure 3) [30].

### Bone

MRI with DWI is increasingly being used to assess bone marrow and metastatic disease, because it is sensitive to bone marrow cell density, the relative proportions of fat and marrow cells, water content, and bone marrow perfusion [31]. In a recently published meta-analysis, the ability of DWI to improve sensitivity for bony metastasis detection was shown to be at the expense of a slight reduction in specificity [32].

**Figure 4:**
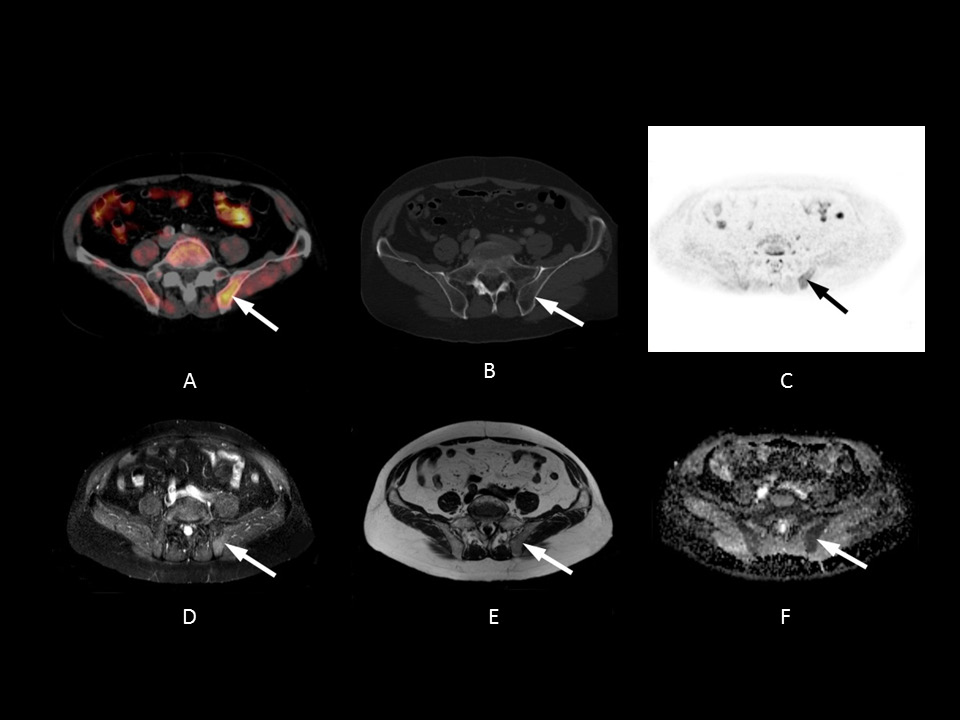
54-year-old patient with bone metastasis in the left pelvis nine years after radical prostatectomy (initial Gleason Score: 8) The PSA level at the time of imaging was 2.3 ng/mL. A fall in PSA level after pelvic irradiation confirmed a malignant lesion (1.0 ng/mL after radiation therapy). **A.** Axial PET/CT fusion image showing a focal 11C-choline uptake in the area of the posterior iliac bone (white arrow, SUV max 3.97). **B.** The lesion shows no substantial sclerosis (HU 708) and is hardly visible on CT. **C.** Axial 500 ms b-image and **D.** ADC map showing only a slight signal abnormality of the lesion in the left iliac bone (relative ADC lesion/normal bone: 1.02) (white and black arrows). **E.** The STIR image shows slight focal signal elevation (relative STIR SI lesion/normal bone: 0.90) and **F.** slight signal distortion can be seen on the T1w image (white arrow, relative SI lesion/normal bone: 1.05).

As a general rule in DWI, however, lytic bony metastases are seen more clearly than pure sclerotic metastases, because of the lower water and cellular content of sclerotic and treated lesions [33]. As a result, DWI may not show purely sclerotic bone metastases very well. In addition, cortical bone, with its very short T2 and long T1 relaxation time, is very poorly interrogated. This might have contributed to our results showing greater accuracy for 11C-choline PET/CT than MRI in recurrent prostate cancer with predominantly sclerotic bone metastases (Figure 4). However, some studies also showed lower 11C-Chloline uptake in sclerotic metastases compared to osteolytic lesions [34]*.* All false negative bone lesions on MRI were located in sites outside of the cervical, thoracic and lumbar spine, with a lower marrow content in relation to cortical structures.

### Limitations

This study has certain limitations. The high mean PSA values indicate a clear selection bias for which a high positive rate was to be expected. Most patients were at high risk of recurrent disease, with a high prevalence of metastases at the time of recurrence. Most of the patients had at least one positive lesion. Thus, the “true” specificity of the methods cannot be determined. A histological examination in all patients would have been optimal but was not possible for practical and ethical reasons.

Instead of a lesion-based analysis of LN metastases, we made region- and patient-based comparisons between imaging and SOR. We chose this approach in consideration of the fact that it is technically extremely challenging to map multiple LNs exactly within a field and correlate each LN with imaging and SOR. However, even this field-wise approach is prone to error when comparing imaging results with follow-up studies.

The use of dedicated sequences for the pelvis, such as DCE imaging, could have improved the accuracy of LR detection. However, we did not use such techniques, in order to stay within a reasonable time frame for whole-body imaging. Significantly more time was needed for MR image acquisition compared to that of PET/CT.

## CONCLUSIONS

11C-choline PET/CT was superior to whole-body MRI with DWI in the detection of local recurrence and bone metastasis on a regional basis. The two methods showed equal diagnostic accuracy for detecting lymph node metastases. In summary, whole-body MRI with DWI cannot be recommended as an alternative to 11C-choline PET/CT. In initial studies, 68Ga-PSMA PET/CT was shown to further enhance the accuracy of PET/CT for recurrent prostate cancer.

## MATERIALS AND METHODS

### Patients

Fifty-seven patients underwent 11C-Choline PET/CT for restaging of PC, which was sort of “routine” in the setting of suspected recurrence of PC during the time of data acquisition. MRI was added as part of our study protocol. Recruitment was performed in an outpatient clinic based on willingness to participate and on the absence of MRI contraindications. During the enrolment period, 10 patients were not eligible for the study because of MRI contraindications (replacements, cardiac devices). Only patients who had undergone radical prostatectomy were eligible for the study. Patients after chemotherapy or after second line hormonal treatment were excluded. No further patient selection was performed to avoid bias and to ensure a broad spectrum of PSA-values. All patients had MRI and 11C-choline PET/CT scans within 1-3 days (mean 1.1 day). For every patient the imaging time needed for 11C-Choline PET/CT and MRI was analyzed (time from scout scan or localizer to the end of acquisition). The mean PSA was 29.9 ng/mL (range 1.0-670 ng/mL). The institutional review board approved the study and all patients gave their informed consent. Table 1 shows the patient characteristics.

### MRI

MRI was performed using a 1.5T MR scanner (Magnetom Avanto, Siemens Medical Solutions, Erlangen, Germany). Two anterior 6-element body matrix coils were used in conjunction with two posterior spine clusters (three channels each) to optimize the signal-to-noise ratio (SNR). In addition, a 12-element head matrix coil, a 4-element neck matrix coil, and a 6-element peripheral matrix coil were used. The maximum imaging range in the z-axis was 160 cm with automated table motion. A total of four axial stacks encompassing the base of the skull to the proximal thighs were acquired. According to the convention derived from various reports in the literature [11, 14, 35], the examination was referred to as a whole-body examination, despite the fact that part of the lower extremities was not included in the imaging field. Axial echo-planar DW sequences including background suppression with spectral selection attenuated inversion recovery (SPAIR) and b-values of 50 and 500 s/mm^2^ were acquired. The selection of a high b-value of 500 s/mm^2^ was based on a trade-off between signal intensity and adequate diffusion strength and was consistent with recommendations given in a consensus paper on the use of DWI in cancer patients [36].

The other technical parameters were as follows: 5 mm slice thickness without intersection gap; number of excitations 3; and generalized autocalibrating partially parallel acquisition (GRAPPA). DWI was achieved by applying pairs of motion-probing gradients before and after the 180° radiofrequency pulse of the spin-echo T2-weighted sequence in three orthogonal directions. ADC maps were calculated from all diffusion weightings and directions on a voxel-by-voxel basis. Conventional T1-weighted (T1w) turbo spin echo (TSE) and short-tau inversion recovery (STIR) images were acquired from the base of the skull to the proximal thigh in axial orientation and for the whole spine in the sagittal plane.

### PET/CT

#### Synthesis of 11C-choline

With minor modifications, 11C-choline was produced according to the method described by Hara et al. [37]. In brief, 11C-CO_2_ was converted to 11C-CH_3_I by the catalytic gas-phase iodination reaction via 11C-CH_4_ (GE MeI MicroLab). 11C-CH_3_I, swept with a helium flow at 50 ml/min, was passed through a Light-CM cartridge loaded with N,N-dimethylethanolamine (25 μl). The column was washed with 10 ml ethanol followed by 10 ml water before eluting the product with isotonic saline (2-5 ml of 0.9% NaCl) through a Millipore filter (Millex GS, 0.22 μm) into a sterile vial. Quality control was performed using HPLC (LiChrosorb RP18, 250×4.6 mm; 1 mM sodium naphthalene sulfonic acid, 50 mM M H_3_PO_4_, 1.5 ml/min; k=3.7).

#### 11C-choline PET/CT

Patients fasted for at least 6 hours before 11C-choline PET. Five minutes after the injection of 600-900 MBq 11C-choline, patients underwent PET/CT (neck to pelvis) in a Siemens Sensation 16 Biograph PET/CT scanner. A diagnostic CT scan was performed prior to PET in the portal venous phase (80 s) after the intravenous injection of contrast agent (Imeron 300) (240 mAs, 120kV, 0.5s per rotation, 5 mm reconstructed slice thickness). All patients received diluted oral contrast (Telebrix 300, 30 ml/L) and rectal filling (100-150 ml). PET scans were acquired in 3D-mode with an acquisition time of 3 minutes per bed position. Iterative reconstruction of the emission data was performed using an ordered-subsets expectation maximization (OSEM) algorithm.

### Image analysis

MRI and 11C-choline PET/CT scans were analyzed independently by two readers (JCS and HW). The readers were blinded to patient data except for the history of prostate cancer with suspected recurrence on basis of PSA elevation. To avoid any learning bias, all datasets were analyzed in random order with an interval of eight weeks between PET and MRI data.

The presence or absence of local recurrence (LR), lymph node (LN), bone or other metastases was evaluated for each patient and imaging modality. As it was not feasible to compare imaging modalities with the standard of reference (SOR) for single LN or bone metastases, we evaluated specific regions as a whole (for bone metastases: cervical, thoracic, and lumbar spine, ribs, right and left sides of the pelvis, upper and lower extremities; for LN metastases: external, internal and common iliac groups on each side, retroperitoneal nodes, supradiaphragmatic nodes). Any other visceral metastases (e.g. in the lungs or liver) were evaluated as one additional region per patient. For each of the anatomical locations, the probability of local recurrence and/or metastases was evaluated using a qualitative 5-point scoring system: definitely present (score 5); probably present (score 4); equivocal (score 3); probably absent (score 2); and definitely absent (score 1). Discordant readings from the two observers were resolved by consensus for each modality.

### PET analysis

Focal 11C-choline activity in the prostatectomy bed greater than adjacent background uptake and not due to radiotracer excreted in the urine was rated 4 or 5, regardless of any corresponding structural abnormality. LNs were rated 4 or 5 if distinct focal activity greater than adjacent background uptake on PET images corresponded to a visible LN on CT, regardless of size; LNs distal to the mid external iliac chains were excluded because increased radiotracer activity in these regions is common [38]. Focal skeletal sites of uptake above background marrow activity were rated 4 or 5, unless explanatory post-traumatic or degenerative changes were evident. The presence of extra-pelvic metastatic lesions was recorded in the same way.

### MRI analysis

Mass-like low T2 signal intensity with diffusion restriction in the prostatectomy bed or in the area of the former neurovascular bundle was taken to be significant for LR and was subsequently rated as 4 or 5. LN involvement was rated on the basis of at least one lymph node per region showing increased signal intensity on inverted b-images: short-axis diameter > 10 mm (score 5), 8-9.9 mm (score 4), 5-7.9 mm (score 3), 1-4.9 mm (score 2), and not seen (score 1). Metastasis to the bone marrow was assumed in focal lesions with an increased signal in the DWI inverted b-image together with signal loss in the corresponding T1w images (lower than muscle).

### Standard of reference (SOR)

The standard of reference was either biopsy and histopathology or clinical follow-up. Histopathology was performed in 14 of the 57 patients (two LR, six LN and six bone metastases). Follow-up was used in 43 of the 57 patients supervised by a tumor board consisting of a nuclear medicine physician, a radiologist, and an urologist. A consensus was obtained using information from prior and subsequent imaging studies and/or clinical data (e.g. start of anti-hormonal therapy or radiotherapy). Follow-up consisted of all the clinical information available after study imaging, including clinical examinations, laboratory tests, radiological procedures, and histopathology from surgical procedures performed at a later date. Five patients died from causes unrelated to prostate cancer during the follow-up period. Minimum follow up time per patient was 24 month (mean 30 months, 24-38 months). Follow-up imaging was done using contrast enhanced CT from thorax-pelvis covering all regions except for the upper and lower extremities. Bone metastases to the extremities were judge in context with other lesions in areas that were covered by CT; there were no solitary or extensive lesions in the extremities that would have required dedicated follow-up imaging. Otherwise judgment of follow-up imaging was based on regions. A significant increase in PSA during follow-up, PSA doubling time < 10 months, confirmation or development of a detectable lesion on follow-up imaging, an increase in lesion size, and a decrease in PSA in response to treatment (irradiation, anti-androgen or chemotherapy) were all considered indicative of malignancy [4, 5].

### Statistics

In order to determine the diagnostic value of each method, receiver operating characteristic curves (ROC) were analyzed on a per region basis. The number of regions detected by MRI and 11C-choline PET/CT were compared with the SOR. Cohen’s kappa was used to determine the agreement between the methods on the basis of score points. Agreement was considered to be slight for κ < 0.21, fair for κ = 0.21-0.40, moderate for κ = 0.41-0.60, substantial for κ = 0.61-0.80, and almost perfect for κ = 0.81-1.00 [39]. Sensitivity, specificity, positive predictive values (PPV), negative predictive values (NPV), and overall predictive accuracy (PAC) were calculated after dichotomization of the score data for each region (score of 4-5: positive for PC lesion; score of 1-3: negative for PC lesion). Comparison of proportions for detecting LR, LN or bone metastases using MRI or 11C-choline PET/CT was performed using the chi-square test. The difference in imaging time for MRI and PET/CT was calculated using the student t-test. A p-value less than 0.05 was considered statistically significant.
